# Understanding the Impact of Social Media Information and Misinformation Producers on Health Information Seeking. Comment on “Health Information Seeking Behaviors on Social Media During the COVID-19 Pandemic Among American Social Networking Site Users: Survey Study”

**DOI:** 10.2196/31415

**Published:** 2022-02-04

**Authors:** Hunter Boudreau, Nikhi Singh, Carter J Boyd

**Affiliations:** 1 School of Medicine University of Alabama at Birmingham Birmingham, AL United States; 2 Hansjörg Wyss Department of Plastic Surgery New York University Langone Health New York, NY United States

**Keywords:** social media, internet, communication, public health, COVID-19, usage, United States, information seeking, web-based health information, online health information, survey, mistrust, vaccination, misinformation

We congratulate Neely and colleagues on their recent work [1] describing the utilization of social media platforms as a source of information regarding the COVID-19 pandemic. The authors suggested that the majority of health information disseminated on social media was not fact-checked with a health care professional [1]. Furthermore, their results demonstrated that subjects following more credible scientific sources on social media were more likely to receive the COVID-19 vaccine [1]. These findings are corroborated by a recent study that concluded that there is a statistically significant relationship between disinformation regarding COVID-19 and lower vaccination rates [2]. However, both studies primarily focused on individual consumers of social media. While these studies are representative samples of the US population, they are unlikely to adequately describe the >200 million Twitter users and are likely subject to selection and recall bias from participants. While analysis of the consumer is revealing, understanding the publishers of information is of equal intrigue and utility.

An alternative methodology for addressing the investigative question proposed by Neely and associates would be to quantify content related to the COVID-19 vaccine and vaccination efforts and further classify this content as informed or misinformed. Data points could include the number of views and the frequency these posts receive subsequent dissemination. This approach would transition the focus from the consumers to the producers of this information. Studies of the aforementioned design come with their own set of limitations; however, we feel it is better suited to address the questions of the authors. Regardless of the study or methodology, social media platforms continue to grow, and health care professionals must recognize the potential effect they can have on social media.

Across social media platforms, it has been previously demonstrated that pro-vaccine individuals are more likely to reference credible sources than those from “antivaccine” groups [3]. Major social media platforms such as Facebook, Twitter, and Instagram have partnered with the World Health Organization in an attempt to target and flag misinformation [3,4]. This served to counter misinformed COVID-19 and other health information on social media. Given social media’s high availability and massive user base, there is a tremendous opportunity for physicians and health care organizations to interact with the American public through these virtual platforms. Establishing a stronger social media presence at both the systems (hospital, national governing medical body, academic center) and individual level is an underutilized opportunity for disseminating health information in an accurate manner. Most physicians (90%) have a presence on social media; however, it is unclear what advocacy impact these accounts have [5]. The introduction of a verification process for posts containing health information may have merit. Implementation of such a policy may increase consumer faith in factual health information, potentially enhancing public health advocacy in campaigns such as COVID-19 vaccinations. It appears that social media has a role to play in health care; an enhanced understanding of social media’s scope of influence and increased physician representation may have a far-reaching impact.
